# A desmoplastic fibroblastoma that developed in the anterior mediastinum: a case report

**DOI:** 10.1186/s13256-021-03014-x

**Published:** 2021-10-18

**Authors:** Tai Hato, Hiroaki Kashimada, Masatoshi Yamaguchi, Ato Sugiyama, Yoshiaki Inoue, Kohei Aoki, Hiroki Fukuda, Masatoshi Gika, Jun Kikuchi, Takashi Fujino, Takehiko Yamaguchi, Jun-ichi Tamaru, Mitsutomo Kohno, Mitsuo Nakayama

**Affiliations:** 1 Department of General Thoracic Surgery, Saitama Medical Center, Saitama Medical University, 1981 Kamoda, Kawagoe, Saitama Japan; 2 Department of Pathology, Saitama Medical Center, Saitama Medical University, 1981 Kamoda, Kawagoe, Saitama Japan; 3 Department of Pathology, Saitama International Center, Saitama Medical University, Hidaka, Saitama Japan; 4 Department of Pathology, Nikko Medical Center, Dokkyo Medical University, Nikko, Tochigi Japan

**Keywords:** Desmoplastic fibroblastoma, Collagenous fibroma, Mediastinal tumor, Video-assisted thoracoscopic surgery, Case report

## Abstract

**Background:**

Desmoplastic fibroblastoma (also known as collagenous fibroma) is a benign, slowly growing soft-tissue tumor. Most desmoplastic fibroblastomas develop in the limbs, neck, or trunk. A mediastinal origin is quite rare.

**Case presentation:**

A 32-year-old Asian female was referred to us for the diagnosis and treatment of an anterior mediastinal tumor. The tumor was 80 mm in the largest diameter and was located on the pericardium. No invasion was evident. She underwent resection of the tumor via video-assisted thoracoscopic resection. The tumor was totally encapsulated, and its pedicle was on the pericardium. The resected specimen was very rigid, making it difficult to remove from the intercostal space. Histologically, the tumor was composed of a paucicellular dense collagenous tissue. Mitosis was rarely observed, and cellular atypia was not evident, suggesting that the tumor was benign. We diagnosed the tumor as a desmoplastic fibroblastoma by morphology and immunohistochemistry.

**Conclusions:**

Desmoplastic fibroblastoma of the mediastinum is an extremely rare disease. Preoperative diagnosis is difficult. Early surgical resection is suitable for diagnosis and treatment planning.

## Background

Desmoplastic fibroblastomas (also known as collagenous fibromas) are benign, collagen-rich soft-tissue tumors. This tumor occurs most frequently in middle-aged adult males. It mainly develops in the limb, neck, and trunk. Histologically, this tumor is characterized by hypocellularity and dense wavy bands of collagenous fibers. This tumor usually appears as a solitary encapsulated mass and grows slowly. Here, we report a rare case of desmoplastic fibroblastoma that arose from the pericardium. Preoperative diagnosis of this tumor was quite difficult. In our case, the tumor size almost doubled within a year, but mitosis was seldomly seen histologically, suggesting that collagen production caused the enlargement of the tumor. To our knowledge, this is the first case of desmoplastic fibroblastoma developed from the pericardium in the literature.

## Case presentation

**Fig. 1:**
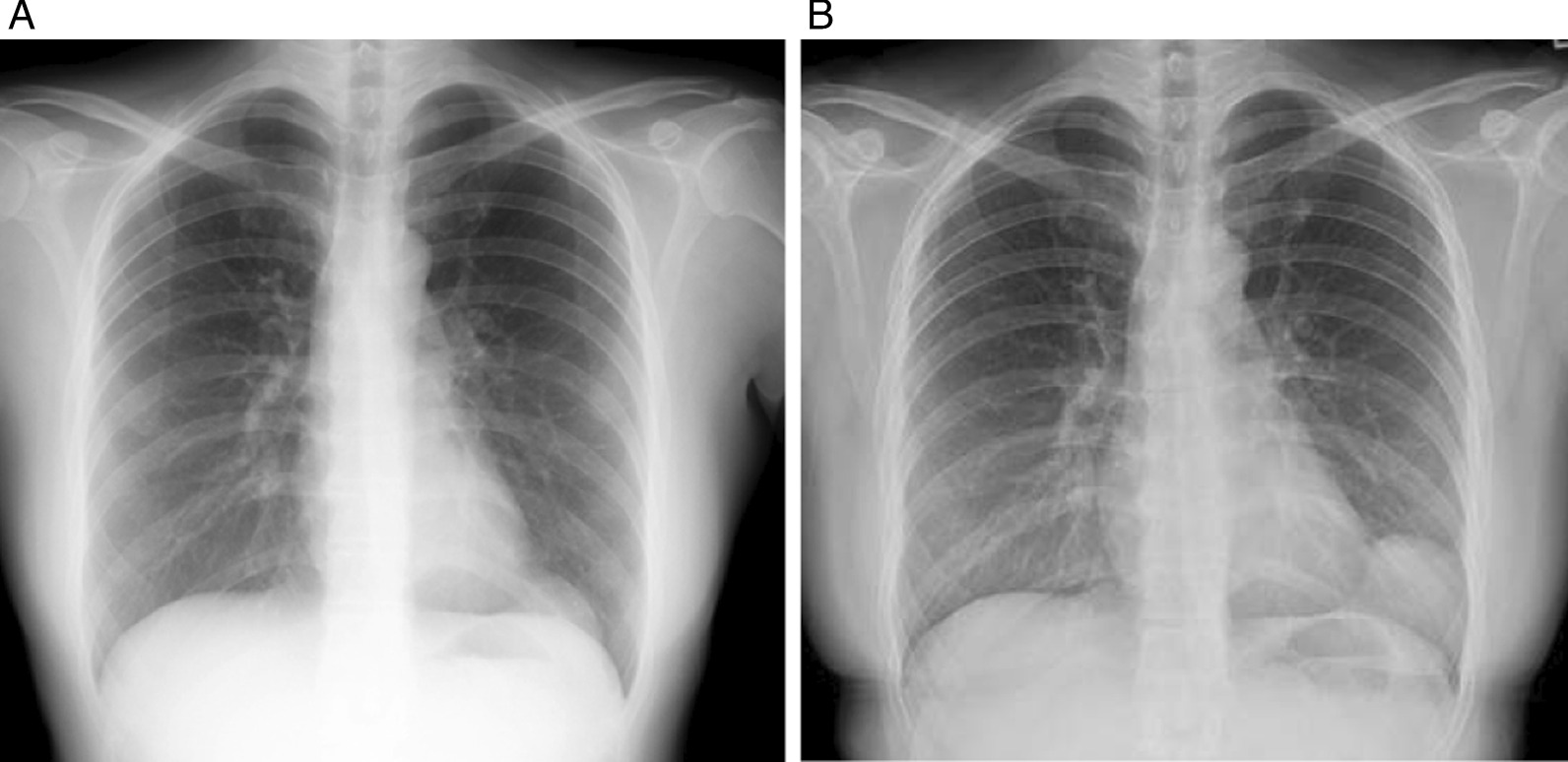
Chest X-ray taken in **A** 2018 and **B** 2019. The tumor size enlarged from 34 to 64 mm in the largest diameter

**Fig. 2:**
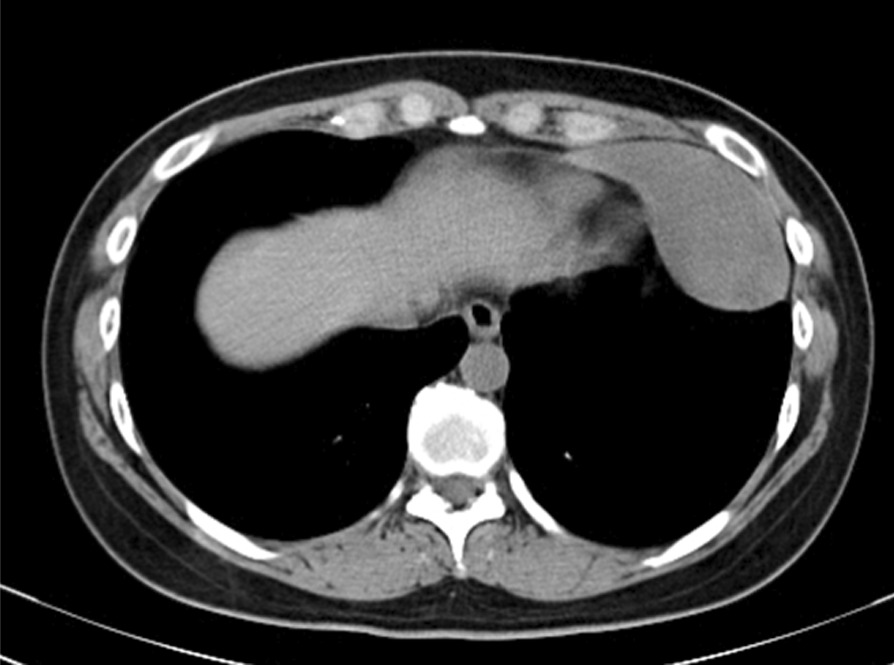
Chest computed tomography image of the tumor. The tumor was located on the pericardium and did not seem to invade the adjacent organs. The tumor had a pedicle on the pericardium

**Fig. 3:**
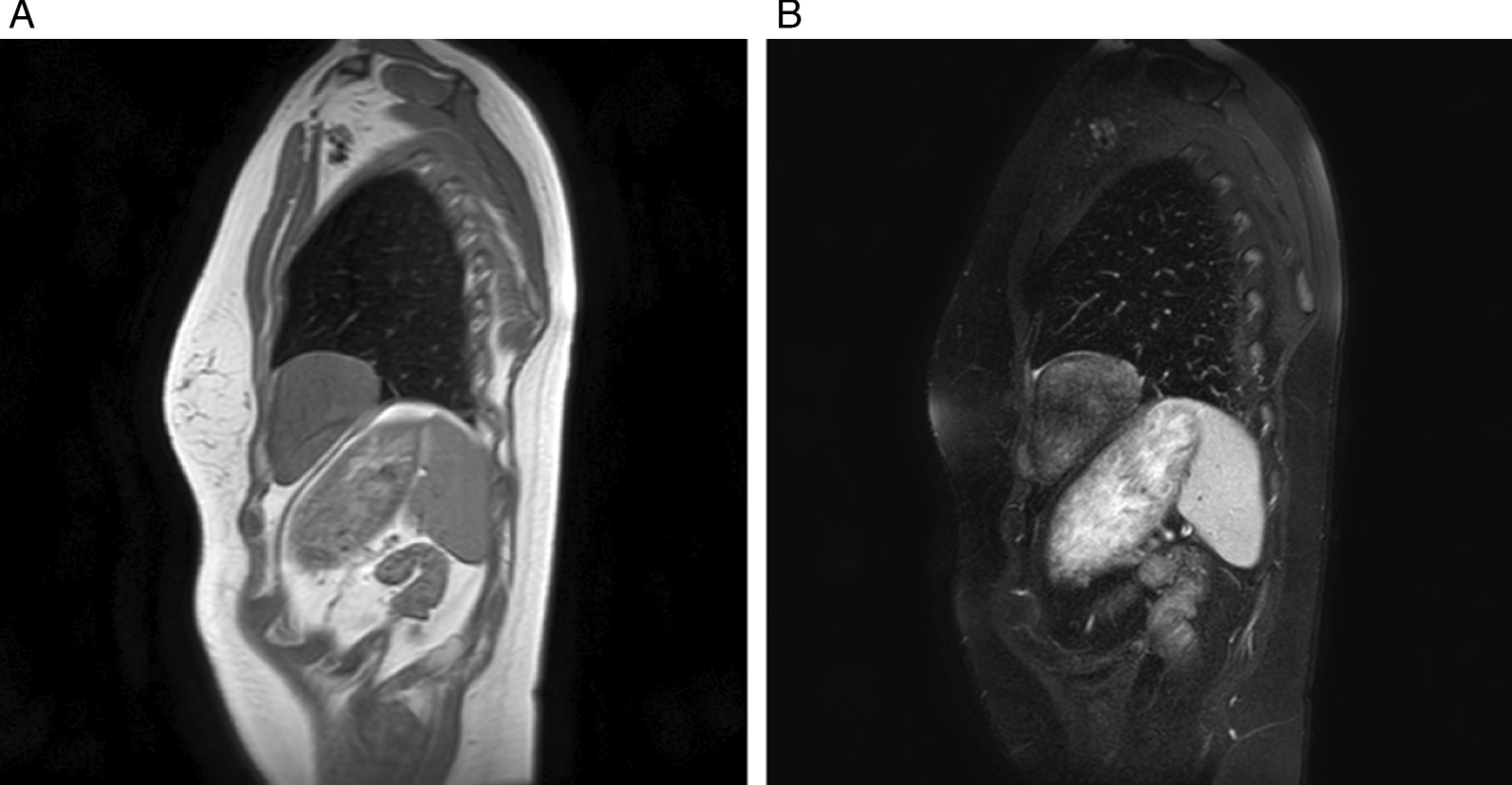
Magnetic resonance images of the tumor. **A** T1-weighted image. **B** T2-weighted fat-saturation image. There was no fat intensity detected inside the tumor

A 32-year-old Asian female visited us for examination of an abnormality on her chest X-ray. There was a well-defined mass lesion around the apex of the heart. The mass had developed and grown within 1 year (Fig. 1A, B). The tumor size enlarged from 34 to 64 mm in the largest diameter by chest X-ray. The patient was asymptomatic, had never smoked, and had a history of hyperthyroidism treated with thiamazole. However, she was medication-free on admission to our hospital. She had no history of pregnancy and no history of childbirth. There was no remarkable family history of illness, including cancer. She is a desk worker with no history of exposure to certain chemicals or asbestos. Her vital signs, physical examination, and neurological examination on admission were unremarkable. Her laboratory finding was within the standard limit on blood cell counts, liver and renal functions, urinalysis, and other serology. Chest computed tomography (CT) revealed a pear-shaped mass lesion measuring 80 mm in the largest diameter that had developed between the pericardium and the left lung (Fig. 2). The lesion was monotonous and had a computed tomography value between 23 and 32 HU. It was growing expansively and did not seem to invade the adjacent organs. The lesion showed a low signal on T1-weighted magnetic resonance imaging (MRI) and a low and heterogeneous signal on T2-weighted fat-saturation imaging (Fig. 3A, B).

**Fig. 4:**
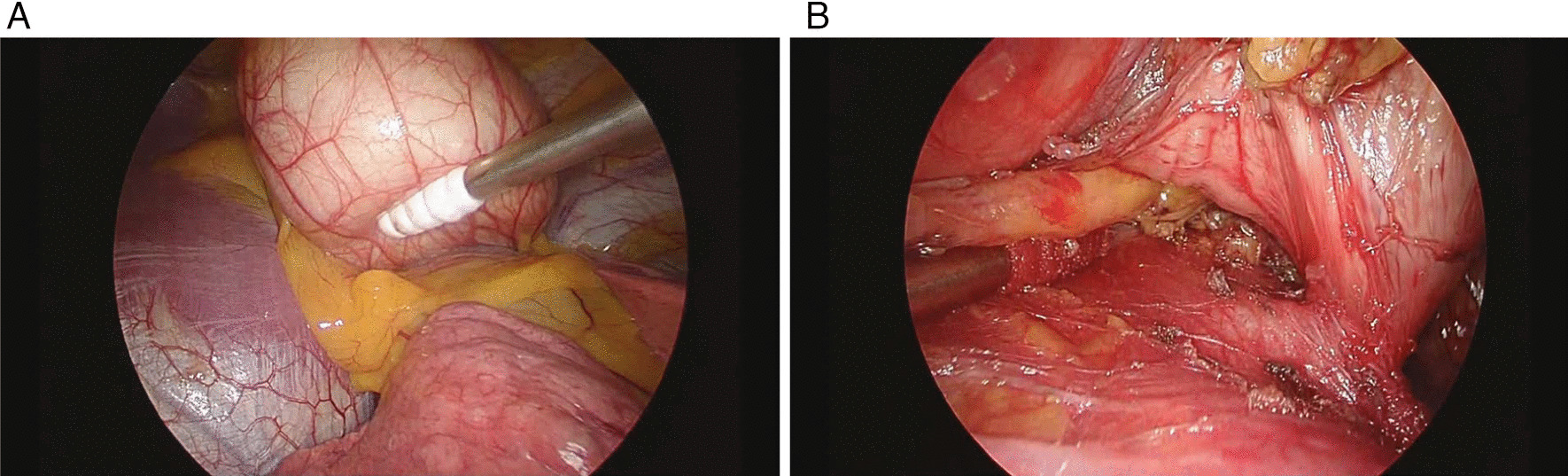
Intraoperative findings. **A** The tumor was located on the pericardium slightly anterior to the phrenic nerve. **B** The tumor was pushed aside with a cotton stick. The pedicle of the tumor was connected to the pericardium

The preoperative diagnosis was a benign anterior mediastinal tumor or a thymoma. Video-assisted thoracoscopic resection of the tumor was performed under general anesthesia. The patient was placed in the right lateral position. An 11.5 mm port was placed in the seventh intercostal space at the lower end of the scapula. Two 5 mm ports were set in the fifth and ninth intercostal spaces on the anterior and posterior axial lines. The tumor was located on the pericardium, slightly anterior to the phrenic nerve (Fig. 4A). It was an encapsulated mass and did not appear to invade the lung or diaphragm. We exfoliated the tumor and fat tissues from the pericardium. The tumor had a pedicle on the pericardium, suggesting its origin (Fig. 4B). After the tumor was resected, we placed it in the specimen bag and attempted to remove it through the largest port. However, the lesion was very rigid, so we could not remove it. We had to enlarge the wound to 35 mm. Her postoperative course was uneventful. She was followed up with computed tomography for 2 years with no evidence of recurrence.

**Fig. 5:**
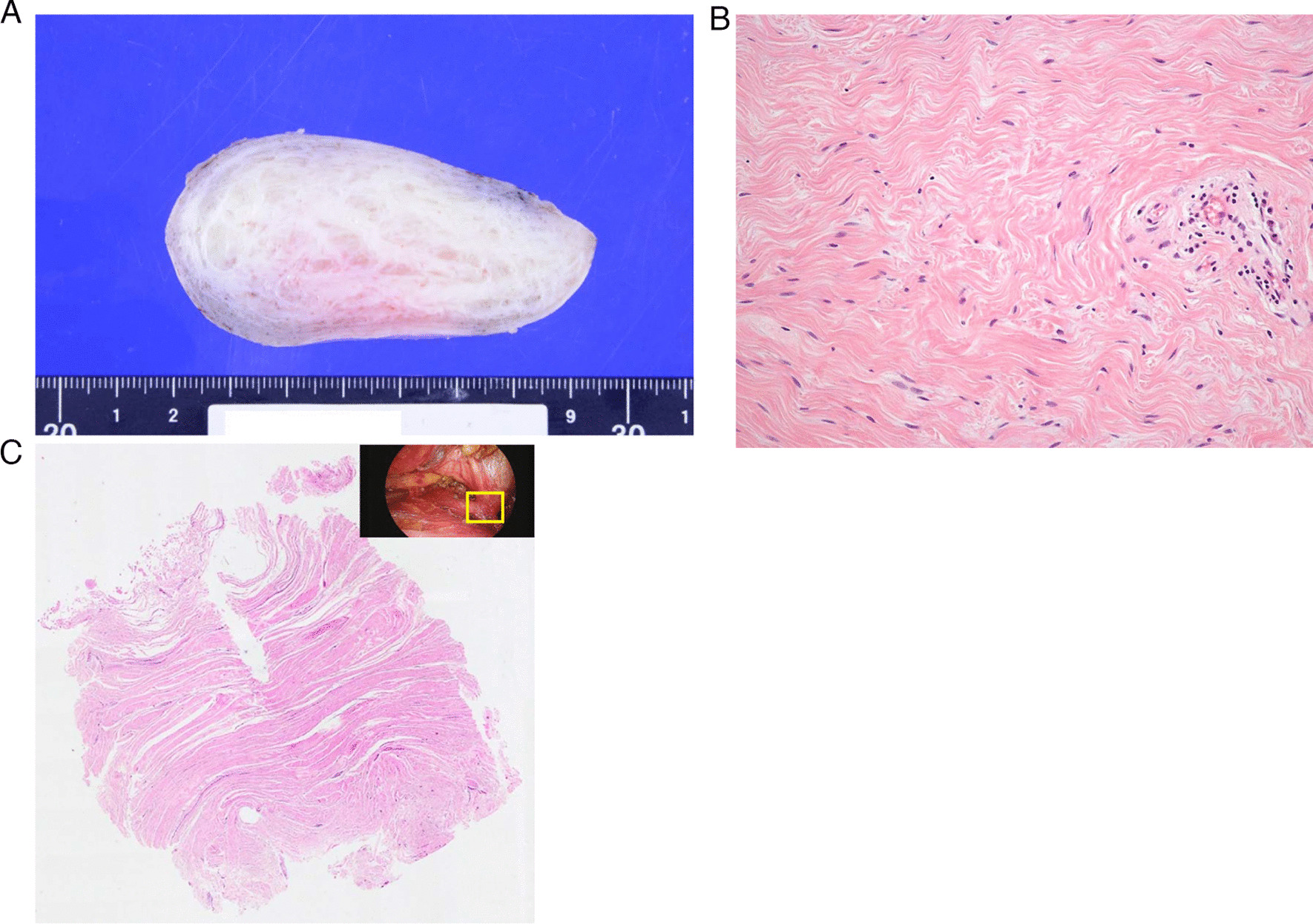
Macroscopic and microscopic findings of the tumor. **A** Gross appearance of the tumor. The cut surface of the tumor showed a dense mesh network of collagen fibers. **B** Hematoxylin–eosin staining showed that the paucicellular tumor was composed of vaguely arranged bundles of wavy collagenous fibers. **C** The pedicle of the tumor was composed of longitudinally arranged fibrous bundles connected to the pericardium, suggesting its origin

The resected tumor was completely encapsulated. There was no invasion to the surrounding pericardial fat or thymus. Macroscopically, the cut surface of the tumor showed a dense mesh network of collagen fibers (Fig. 5A). Hematoxylin–eosin staining showed that the paucicellular tumor was composed of vaguely arranged bundles of wavy collagen fibers (Fig. 5B). The pedicle of the tumor was composed of longitudinally arranged fibrous bundles connected to the pericardium, suggesting its origin (Fig. 5C). The cellularity was sparse. The tumor cells did not seem to be atypical. Some nonspecific lymphocyte (or mononuclear cell) infiltration could be seen around the tumor vasculature with different intensities. Immunohistochemical analysis showed a positive reaction for alpha-smooth muscle actin (αSMA), desmin, and CD34. Integrase interactor 1 (INI1) expression was intact. Staining for cytokeratin (AE1/AE3), epithelial membrane antigen (EMA), β-catenin, S100 protein, D2-40, MDM2, and STAT6 was negative. There was no infiltration of IgG4-positive plasma cells. These results suggested fibroblastic or myofibroblastic differentiation. The Ki-67 labeling index was less than 5%. Anaplastic leukemia kinase (ALK) staining was negative on immunohistochemistry. Fluorescent *in situ* hybridization indicated no breakage of the ALK gene. Finally, the tumor was diagnosed as desmoplastic fibroblastoma.

## Discussion

We report a rare case of desmoplastic fibroblastoma originating from the pericardium. To the best of our knowledge, our study is the first report of a tumor developed in the anterior mediastinum.

Desmoplastic fibroblastoma (also known as collagenous fibroma) is a benign, slow-growing soft-tissue tumor. Evans *et al*. reported the first case in 1995 [1]. This tumor occurs mainly in middle-aged or older men [2]. Most of these tumors arise in subcutaneous tissues, the fascia, and skeletal muscles. Common sites of origin include the limbs, neck, and trunk [2, 3]. However, rarer sites of origin have also been reported, including the palate, parotid glands, and thyroid glands [4–6]. One case involving a chest wall origin was reported, but a pericardial origin is quite rare [7].

The preoperative diagnosis was difficult. Computed tomography showed a uniform tumor connected to the pericardium. The computed tomography value of the tumor and the MRI findings excluded the probability of cystic disease. Imaging differential diagnosis included diverse kinds of soft-tissue tumors and thymic epithelial tumors. However, the pear shape of the tumor is not typical of thymoma. Both CT and MRI showed no fat component inside the tumor, so lipoma and liposarcoma were ruled out. Due to the rarity of soft-tissue tumors of the mediastinum, further diagnosis by imaging alone was difficult.

Interestingly, the tumor grew almost double in size within a year. However, mitosis was seldom seen microscopically. This would suggest that the tumor produced abundant fibrous tissues that mimicked tumor growth. Because the collagenous fibers were quite dense, we could not shape or compress the tumor to extract it through the intercostal space.

The histologic differential diagnosis included desmoid-type fibromatosis, solitary fibrous tumor, sclerosing rhabdomyosarcoma, and inflammatory myofibroblastic tumor. The absence of β-catenin immunostaining and the morphology did not match desmoid-type fibromatosis. Monotonous histology, lack of the pericytomatous pattern of the vasculature, and negativity of STAT6 staining excluded the possibility of solitary fibrous tumor. The lack of atypia and mitosis did not match the features of rhabdomyosarcoma. Neither ALK staining on immunohistochemistry nor breakage of the ALK gene on fluorescent *in situ* hybridization was detected. Therefore, the probability of an inflammatory myofibroblastic tumor was also low. Finally, the tumor was diagnosed as desmoplastic fibroblastoma. It has been reported that immunohistochemical studies are of little help in making a diagnosis of desmoplastic fibroblastoma because of the lack of tumor-specific markers [8]. Nevertheless, immunohistochemical examinations are useful for exclusionary diagnosis. The limitation of this case report is the unavailability of cytogenetic analysis. Cytogenetic analysis describing a chromosomal rearrangement in 11q12 and translocation(2;11) has been reported in the diagnosis of desmoplastic fibroblastoma [3]. However, an *in situ* hybridization probe was not available for this case. Instead, we attempted whole-genome sequencing for this tumor. Unfortunately, it was impossible because the quality and amount of DNA from the formalin-fixed paraffin-embedded samples was insufficient. DNA sampling was impossible due to the desmoplastic nature and scant cellularity of this tumor.

## Conclusions

We report the first case of desmoplastic fibroblastoma arising from the pericardium. A preoperative diagnosis of this kind of tumor is almost impossible. The tumor grows quickly, but little mitosis is seen in histology. Surgical resection is suitable for the diagnosis and cure of this benign disease.

## Data Availability

All data used and analyzed during the current study are available from the corresponding author on reasonable request.
